# Fox’s Atlas and Text-Book of Skin Diseases

**Published:** 1888-03

**Authors:** 


### BOOK NOTICES.

FOX'S ATLAS AND TEXT-BOOK OF SKIN DISEASES. Second Edition. Issued in parts by E. B. Treat & Co., New York, 1888.

It is scarcely necessary for us to do more than mention this standard work, for both the author and the work are far too well known to make any criticism needed. The plates are certainly most excellently well done. The new edition is a text book as well as an atlas of skin diseases. There are to be twelve parts at two dollars each, with four colored plates in each part.

